# Editorial: Precision strategies for atrial fibrillation: diagnosis, risk, and treatment innovations

**DOI:** 10.3389/fcvm.2026.2028805

**Published:** 2026-10-30

**Authors:** Ioannis Anagnostopoulos, Giulio Stefanini, Dimitrios Vrachatis

**Affiliations:** 1 Electrophysiology and Catheterization Laboratory, Eugenideio Clinic, National and Kapodistrian University of Athens, Athens, Greece; 2 IRCCS Humanitas Research Hospital, Rozzano, Lombardy, Italy; 3 Department of Biomedical Sciences, Humanitas University, Milan, Lombardy, Italy; 4 Department of Biomedical Engineering, Medical School, National and Kapodistrian University of Athens, Athens, Greece

**Keywords:** AF-CARE, artificial intelligence, atrial fibrillation, biomarkers, catheter ablation, precision medicine, risk stratification, wearables

## Introduction

Atrial fibrillation (AF) is the most common sustained arrhythmia worldwide, and its burden grows as populations age and cardiometabolic disease becomes more prevalent. The 2024 European Society of Cardiology (ESC) guidelines organize AF management around the AF-CARE framework: Comorbidity and risk-factor management; Avoidance of stroke and thromboembolism; Reduction of symptoms by rate and rhythm control; and Evaluation with dynamic reassessment 1. AF-CARE is patient-centered by design, but each pillar still relies largely on population-derived tools. This Research Topic compiles studies that could make these tools more personalized. Its 34 contributions span mechanisms, epidemiology, imaging, artificial intelligence (AI), and procedural innovation; we summarize them along the AF-CARE pathway (Figure 1).

**Figure 1:**
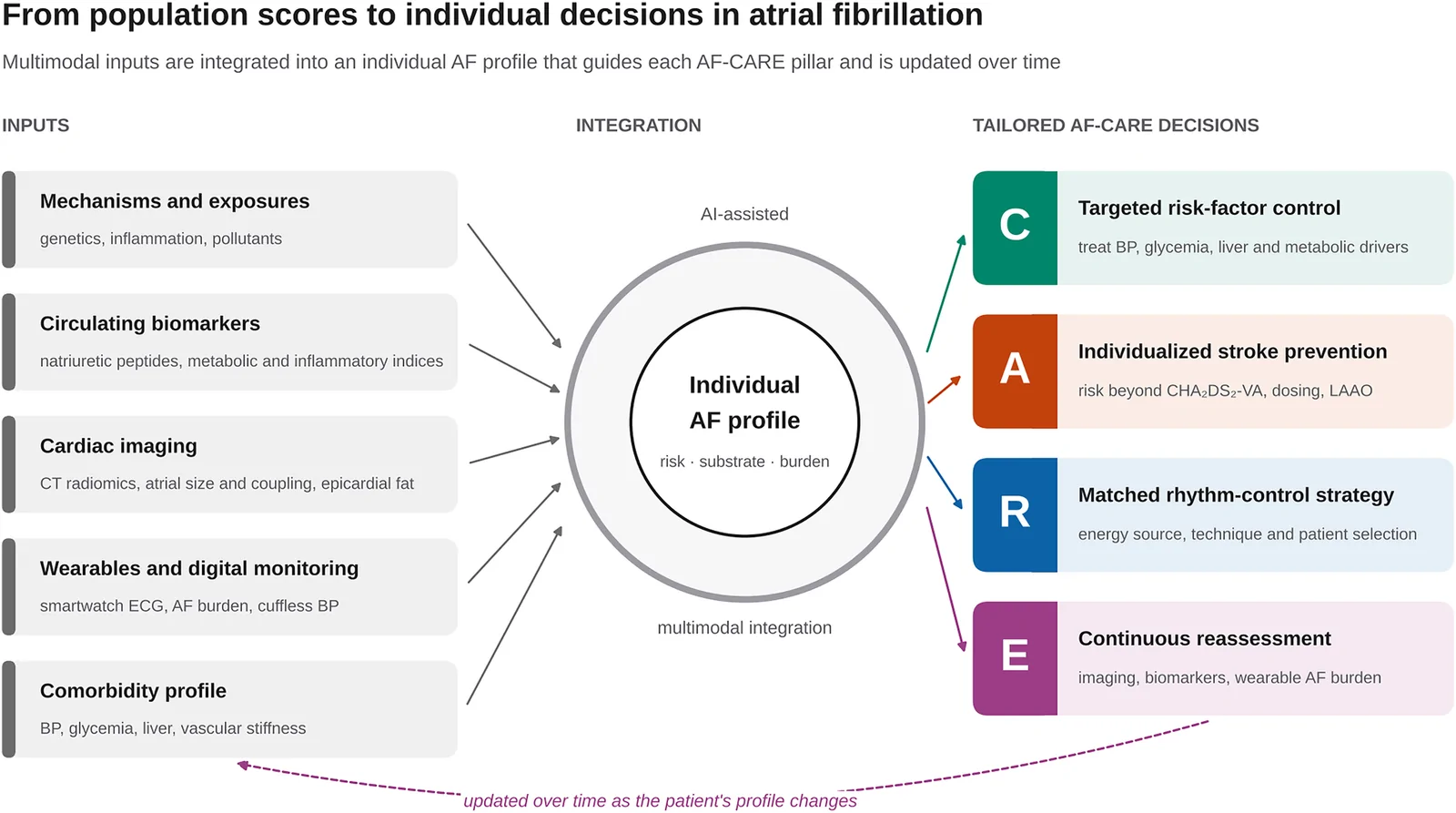
Conceptual model of precision management in atrial fibrillation. Mechanistic, biomarker, imaging, wearable, and comorbidity data, as studied in this Research Topic, are increasingly integrated, with AI support, into an individual AF profile that guides each pillar of the 2024 ESC AF-CARE framework 1 and is updated over time. AF, atrial fibrillation; AI, artificial intelligence; BP, blood pressure; CT, computed tomography; ECG, electrocardiogram; ESC, European Society of Cardiology; LAAO, left atrial appendage occlusion.

### Foundations: mechanisms and substrate

Li et al. combined network toxicology, Mendelian randomization, and cell experiments to propose complement factor B as a link between the pollutant perfluorooctanoic acid and AF-related molecular changes. Ren et al. reviewed how macrophage phenotypes drive electrical and structural remodeling. Circulating signals were also explored: impaired thyroid hormone sensitivity was associated with persistent AF in euthyroid patients (Gong et al.), serum metabolomics identified candidate early-warning metabolites in hyperuricemia (Li et al.), and vascular adhesion protein-1 was associated with AF and adverse events (Zhang et al.).

### C: comorbidity and risk-factor management

Che et al. found a linear association between admission blood pressure and in-hospital stroke in more than 25,000 hospitalized patients. Zhao et al. described a bidirectional relationship between arterial stiffness and AF. A meta-analysis of over 15 million participants linked prediabetes to a 20% higher AF risk (Li et al.), moving the preventive window earlier. Composite markers also proved informative: the uric acid-to-albumin ratio predicted mortality beyond CHA_2_DS_2_-VASc in two independent cohorts (Gong et al.), and a higher prognostic nutritional index was associated with fewer adverse events in AF with heart failure and preserved ejection fraction (Xu et al.).

### A: avoiding stroke and thromboembolism

Gao et al. developed interpretable machine-learning models for patients with CHA_2_DS_2_-VA scores of 1 or less. Anticoagulation decisions were found to be most uncertain in this subpopulation; NT-proBNP was the most influential predictor. Ratajczak et al. used echocardiographic and P-wave features to identify left atrial thrombus despite continuous anticoagulation, and Nagy et al. reviewed the limited evidence on thrombus persistence with NOAC therapy. Prescribing represents a further gap: more than half of hospitalized patients in one cohort received an inappropriate DOAC dose, largely due to underdosing (Bai et al.). Ren et al. found comparable sealing across three occluders for appendage occlusion combined with ablation, and Zhang et al. described a one-stop procedure for cor triatriatum sinister.

### R: reducing symptoms by rate and rhythm control

A network meta-analysis ranked pulsed field ablation (PFA) as the most effective treatment for freedom from atrial arrhythmia and shortest procedure time (Feng et al.). High-power, short-duration radiofrequency shortened procedure time without increasing the risk of complications (He et al.). A PFA review (Sun et al.), a thermometry-guided cryoballoon that reduces procedure time, radiation, and contrast (Bai et al.), along with multifrequency impedance mapping of ablated tissue (Amorós-Figueras et al.) illustrated continuing technical refinement. In typical flutter, a continuous dragging technique achieved a higher first-pass block rate than point-by-point ablation (Turcsan et al.); during rheumatic mitral valve surgery, cryoablation produced greater left atrial reverse remodeling than radiofrequency ablation (Nurbay et al.). Patient selection matters as much as technique: fatty liver disease (Xu et al.) and inflammation combined with poor blood pressure control (Wei et al.) were found to predict recurrence, whereas patients whose AF was detected after a stroke fared similarly to those with known AF (Liu et al.).

### E: evaluation and dynamic reassessment

Imaging, biomarkers, and wearables could support reassessment over time. Using cardiac computed tomography, radiomics with fractal dimension predicted recurrence after ablation (Li et al.), the left atrioventricular coupling index outperformed left atrial volume (Du et al.), and epicardial fat distinguished persistent from paroxysmal AF in patients with reduced ejection fraction (Ma et al.). Lower secretoneurin levels after ablation were associated with recurrence (Plášek et al.) and, unexpectedly, with higher cuffless nocturnal blood pressure variability with fewer recurrences (Murota et al.). Smartwatch-derived AF burden, unlike binary recurrence, tracked quality-of-life gains after ablation (Almeida et al.), and AI applied to wearable ECG predicted the direction of NT-proBNP change (Song et al.). Machine learning also predicted new-onset AF in acute respiratory failure (Li).

### Outlook

The articles presented in this Special issue denote that precision in AF is moving from a concept to measurable tools at every stage of AF-CARE. However, the majority of studies were retrospective and single-center; the cohorts came largely from one region; and several machine-learning models lacked external validation. Prospective, multicenter validation in diverse populations, ideally within trials that use these tools to guide therapy, is the essential next step.

More than a century after its first electrocardiographic description, the mechanisms that initiate and sustain AF remain incompletely understood. This Research Topic reinforces a view of AF not as a single disease, but rather as the common expression of diverse substrates (inflammatory, metabolic, hemodynamic, structural, and environmental) that differ between patients and evolve within each patient over time. Cardiology has made this conceptual shift before: coronary artery disease is now approached as a spectrum of coronary syndromes, with heterogeneous mechanisms and a holistic, multidisciplinary model of care. AF deserves the same reframing. Treating AF as a syndrome, with phenotypes defined by mechanism rather than by episode duration alone and therapy matched to each phenotype, may be the decisive step toward truly precise care. AF-CARE provides the scaffold; the task ahead is to build each patient into it.
